# Supplementary material for: The Evolution and Characterization of the RNA Interference Pathways in Lophotrochozoa
Source: Genome Biol Evol. 2024 May 7;16(5):evae098. doi: 10.1093/gbe/evae098 (PMC11114477; doi:10.1093/gbe/evae098)
Supplement: evae098_Supplementary_Data [file evae098_supplementary_data.zip › Supplementary_materials.zip!/selected_miRNAs/NoGe_selected_miRNAs/mirdeep_pdf_files/NMRB01000451.1_Notospermus_geniculatus_strain_Ushimado_scaffold451,_whole_genome_shotgun_sequence_13662.pdf]

Provisional ID	:	NMRB01000451.1_Notospermus_geniculatus_strain_Ushimado_scaffold451,_whole_genome_shotgun_sequence_13662
Score total	:	133114.1
Score for star read(s)	:	3.9
Score for read counts	:	133105.8
Score for mfe	:	1.4
Score for randfold	:	0
Score for cons. seed	:	3
Total read count	:	261093
Mature read count	:	260959
Loop read count	:	0
Star read count	:	134

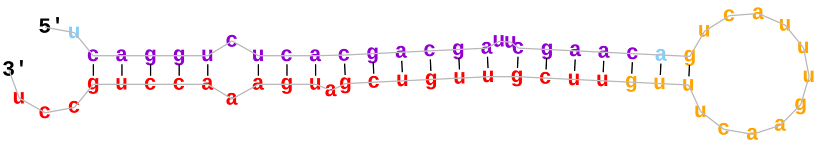

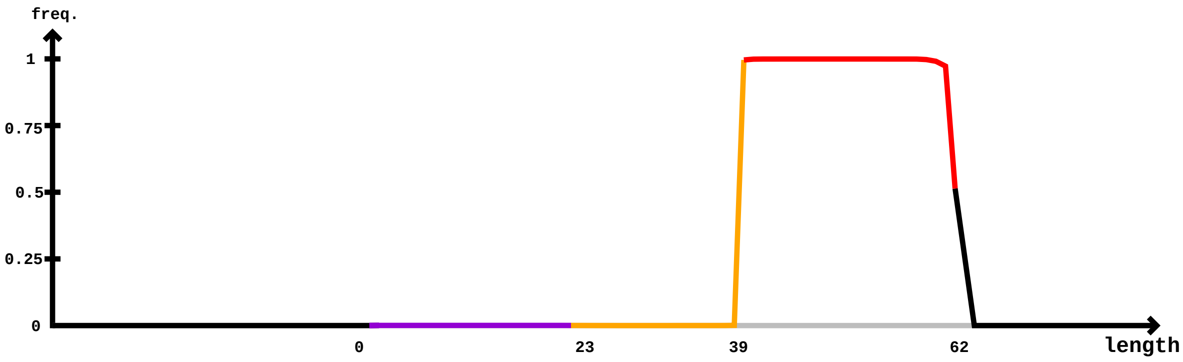

Star Mature

```
5'- ccucuauuagaaggaaucaggacauccaaauucaggucucacgacgauucgaacagucauuugaacuuuguucguugucgaugaaaccugccugcugguguccgagucgucga   -3'    obs
    ccucuauuagaaggaaucaggacauccaaauucaggucucacgacgauucgaacagucauuugaacuuuguucguugucgaugaaaccugccugcugguguccgagucgucga          exp
    .........((..((.((.(((((((......(((((.(((((((((..(((((((...........))))))))))))).))).)))))......))))))))).)).))..    reads    mm    sample
    ...............................ucaggucucacgacgauucga.............................................................    6    0    Fm3
    ...............................ucaggucucacgacgauucgaac...........................................................    2    0    Fm3
    ...............................ucaggucucacgacgauucgaacaguc.......................................................    2    0    Fm3
    ................................caggucucacgacgauucg..............................................................    2    0    Fm3
    ................................caggucucacgacgauucga.............................................................    10    0    Fm3
    ................................caggucucacgacgauucgaac...........................................................    29    0    Fm3
    ................................caggucucacgacgauucgaacU..........................................................    3    1    Fm3
    ................................caggucucacgacgauucgaaca..........................................................    9    0    Fm3
    ................................caggucucacgacgauucgaacagu........................................................    3    0    Fm3
    ................................caggucucacgacgauucgaacagucauu....................................................    2    0    Fm3
    ................................................................acuuuguucguugucgaugaaaccu........................    1    0    Fm3
    .................................................................cuuuguucguugucgaugaa............................    6    0    Fm3
    .....................................................................guucguugucgaugaaaccu........................    2    0    Fm3
    .....................................................................guucguugucgaugaaaccug.......................    1    0    Fm3
    .....................................................................Cuucguugucgaugaaaccugcc.....................    3    1    Fm3
    .....................................................................guucguugucgaugaaaccugcc.....................    2    0    Fm3
    .....................................................................guucguugucgaugaaaccugccu....................    1    0    Fm3
    .....................................................................Cuucguugucgaugaaaccugccu....................    8    1    Fm3
    .....................................................................Auucguugucgaugaaaccugccu....................    1    1    Fm3
    ......................................................................uucguugucgaugaaacA.........................    1    1    Fm3
    ......................................................................uucguugucgaugaaacc.........................    17    0    Fm3
    ......................................................................uucguugucgaugaaacU.........................    3    1    Fm3
    ......................................................................uucguugucgaugaaacAu........................    2    1    Fm3
    ......................................................................uucguugucgaugaaaAcu........................    2    1    Fm3
    ......................................................................uucguugucgaugaaaGcu........................    1    1    Fm3
    ......................................................................uucguuUucgaugaaaccu........................    1    1    Fm3
    ......................................................................uucguugucgaugaaacUu........................    3    1    Fm3
    ......................................................................uucguugucgaugaaaccu........................    250    0    Fm3
    ......................................................................uucguuguUgaugaaaccu........................    1    1    Fm3
    ......................................................................uucguugucgGugaaaccu........................    1    1    Fm3
    ......................................................................uucguugucgaugaaaccug.......................    598    0    Fm3
    ......................................................................uucguugucgaugCaaccug.......................    1    1    Fm3
    ......................................................................uucguugucgaugaaacGug.......................    2    1    Fm3
```

```
                                         Star                                          Mature
ccucuauuagaaggaaucaggacauccaaauucaggucucacgacgauucgaacagucauuugaacuuuguucguugucgaugaaaccugccugcugguguccgagucgucga
......................................................................uucguugucgaugaaaGcug.......................	2	1	Fm3
......................................................................uucguugucgCugaaaccug.......................	1	1	Fm3
......................................................................uucguugucgaugaaacAug.......................	8	1	Fm3
......................................................................uucguugucgaugaaacUug.......................	1	1	Fm3
......................................................................uucguugucgaugaaaAcug.......................	3	1	Fm3
......................................................................uucguugucgGugaaaccug.......................	3	1	Fm3
......................................................................uucguugucCaugaaaccug.......................	1	1	Fm3
......................................................................uucguugucgaugaaaccugc......................	1064	0	Fm3
......................................................................uucguugucgaugaGaccugc......................	1	1	Fm3
......................................................................uucguugucgaugaaacUugc......................	5	1	Fm3
......................................................................uucguugucgaugaaacAugc......................	12	1	Fm3
......................................................................uucguugucgaugaaacGugc......................	5	1	Fm3
......................................................................uuAguugucgaugaaaccugc......................	1	1	Fm3
......................................................................uucguugucgaugaaaAcugc......................	2	1	Fm3
......................................................................uucguugucAaugaaaccugc......................	1	1	Fm3
......................................................................uucguugucgaugaaaccuCc......................	1	1	Fm3
......................................................................uucguugucgaugaaaccugA......................	3	1	Fm3
......................................................................uucguugucgaugaaaccugU......................	107	1	Fm3
......................................................................uucguugucgaugaaaUcugc......................	3	1	Fm3
......................................................................uucguugucgaugaaaGcugc......................	2	1	Fm3
......................................................................uucAuugucgaugaaaccugc......................	1	1	Fm3
......................................................................uucguugucgaugUaaccugc......................	1	1	Fm3
......................................................................uucguugucgaCgaaaccugcc.....................	2	1	Fm3
......................................................................uucguugucgaAgaaaccugcc.....................	1	1	Fm3
......................................................................uucguCgucgaugaaaccugcc.....................	1	1	Fm3
......................................................................uucguuAucgaugaaaccugcc.....................	3	1	Fm3
......................................................................uucguugucgaugaaaccCgcc.....................	1	1	Fm3
......................................................................uucgAugucgaugaaaccugcc.....................	3	1	Fm3
......................................................................uucguGgucgaugaaaccugcc.....................	6	1	Fm3
......................................................................uucguuCucgaugaaaccugcc.....................	1	1	Fm3
......................................................................uucguugucUaugaaaccugcc.....................	3	1	Fm3
......................................................................uucguugucgaugUaaccugcc.....................	1	1	Fm3
......................................................................uucguugucgauUaaaccugcc.....................	1	1	Fm3
......................................................................uucAuugucgaugaaaccugcc.....................	5	1	Fm3
......................................................................uAcguugucgaugaaaccugcc.....................	7	1	Fm3
......................................................................uucguugucAaugaaaccugcc.....................	3	1	Fm3
......................................................................uucguugucgauAaaaccugcc.....................	1	1	Fm3
......................................................................uucguugucgaugaUaccugcc.....................	2	1	Fm3
......................................................................uucguugucgaugaaaccuCcc.....................	5	1	Fm3
......................................................................uucguugucgaugaaacUugcc.....................	143	1	Fm3
......................................................................uucguuguUgaugaaaccugcc.....................	11	1	Fm3
......................................................................uucguugucgaugaaaGcugcc.....................	42	1	Fm3
......................................................................uucguugucgaugaaaAcugcc.....................	86	1	Fm3
......................................................................uucguugucgaugaaaUcugcc.....................	71	1	Fm3
......................................................................uCcguugucgaugaaaccugcc.....................	3	1	Fm3
......................................................................uucguugucgCugaaaccugcc.....................	32	1	Fm3
......................................................................uucguuguGgaugaaaccugcc.....................	1	1	Fm3
......................................................................uuUguugucgaugaaaccugcc.....................	4	1	Fm3
......................................................................uucguugucgaugaaacAugcc.....................	215	1	Fm3
......................................................................uucguugucgGugaaaccugcc.....................	33	1	Fm3
......................................................................uucguuguAgaugaaaccugcc.....................	3	1	Fm3
......................................................................uucguugucCaugaaaccugcc.....................	1	1	Fm3
......................................................................uucCuugucgaugaaaccugcc.....................	1	1	Fm3
......................................................................Aucguugucgaugaaaccugcc.....................	4	1	Fm3
......................................................................uucguugucgaugaaaccugGc.....................	1	1	Fm3
......................................................................uucgGugucgaugaaaccugcc.....................	2	1	Fm3
......................................................................uucguugucgaugaaaccuUcc.....................	14	1	Fm3
......................................................................uuGguugucgaugaaaccugcc.....................	5	1	Fm3
......................................................................Cucguugucgaugaaaccugcc.....................	7	1	Fm3
......................................................................uucUuugucgaugaaaccugcc.....................	4	1	Fm3
......................................................................uucguugucgaugGaaccugcc.....................	3	1	Fm3
......................................................................uucguugucgaugaaaccugcc.....................	21966	0	Fm3
......................................................................uucguugAcgaugaaaccugcc.....................	1	1	Fm3
......................................................................uucguugucgaugCaaccugcc.....................	2	1	Fm3
......................................................................uucguugucgaugaaaccGgcc.....................	1	1	Fm3
......................................................................uucguNgucgaugaaaccugcc.....................	1	1	Fm3
......................................................................uGcguugucgaugaaaccugcc.....................	2	1	Fm3
......................................................................uuAguugucgaugaaaccugcc.....................	5	1	Fm3
......................................................................Nucguugucgaugaaaccugcc.....................	12	1	Fm3
......................................................................uucguugucgUugaaaccugcc.....................	23	1	Fm3
```

```
                                  Star                       Mature
ccucuauuagaaggaaucaggacauccaaauucaggucucacgacgauucgaacagucauuugaacuuuguucguugucgaugaaaccugccugcuggugucgagucgucga
......................................................................uucguugucgaugaaaccugcU....................    302     1     Fm3
......................................................................uucguugucgaugaGaccugcc....................    2       1     Fm3
......................................................................uucguuNucgaugaaaccugcc....................    2       1     Fm3
......................................................................uucguugucgaugaaaccugAc....................    5       1     Fm3
......................................................................uucgCugucgaugaaaccugcc....................    5       1     Fm3
......................................................................uucguugucgaugaaUccugcc....................    2       1     Fm3
......................................................................uucguugucgaugaaaccugUc....................    11      1     Fm3
......................................................................uucguugucgaugaaaccugcA....................    9       1     Fm3
......................................................................uucguugucgaugaaacGugcc....................    90      1     Fm3
......................................................................uucguugucgauCaaaccugcc....................    4       1     Fm3
......................................................................uucguuUucgaugaaaccugcc....................    7       1     Fm3
......................................................................uucguugucgaugaaaccuAcc....................    8       1     Fm3
......................................................................uucguugAcgaugaaaccugccu...................    1       1     Fm3
......................................................................uucguugucgGugaaaccugccu...................    23      1     Fm3
......................................................................uucguugucUaugaaaccugccu...................    1       1     Fm3
......................................................................uucguugucgaugaaaccugccA...................    162     1     Fm3
......................................................................uucguugucgaugaaaccCgccu...................    4       1     Fm3
......................................................................uucguugucgaugaaaccugGcu...................    3       1     Fm3
......................................................................uCcguugucgaugaaaccugccu...................    2       1     Fm3
......................................................................uucguugucgaugaaaccugcUu...................    25      1     Fm3
......................................................................uucguugucgaugGaaccugccu...................    5       1     Fm3
......................................................................uuAguugucgaugaaaccugccu...................    4       1     Fm3
......................................................................uucguugucgauAaaaccugccu...................    5       1     Fm3
......................................................................uucguugucgaGgaaaccugccu...................    1       1     Fm3
......................................................................uucguuguAgaugaaaccugccu...................    2       1     Fm3
......................................................................uucguuNucgaugaaaccugccu...................    2       1     Fm3
......................................................................uucguugucAaugaaaccugccu...................    4       1     Fm3
......................................................................uucguugucgaAgaaaccugccu...................    1       1     Fm3
......................................................................uucguugucgaugaCaccugccu...................    2       1     Fm3
......................................................................uucguugucgaugaaacAugccu...................    250     1     Fm3
......................................................................uucguugucgaugUaaccugccu...................    6       1     Fm3
......................................................................uucguNgucgaugaaaccugccu...................    2       1     Fm3
......................................................................uucguugucgaugaaaccuCccu...................    2       1     Fm3
......................................................................uuGguugucgaugaaaccugccu...................    5       1     Fm3
......................................................................uucguugucgaugaaacGugccu...................    116     1     Fm3
......................................................................uucguuguUgaugaaaccugccu...................    20      1     Fm3
......................................................................uucguugucgaugaaaccuAccu...................    11      1     Fm3
......................................................................uucguugucgaugaaaccugccG...................    1       1     Fm3
......................................................................uucgCugucgaugaaaccugccu...................    2       1     Fm3
......................................................................uucguGgucgaugaaaccugccu...................    3       1     Fm3
......................................................................uucguugCcgaugaaaccugccu...................    3       1     Fm3
......................................................................uucCuugucgaugaaaccugccu...................    3       1     Fm3
......................................................................uucguugucgaugaaaAcugccu...................    116     1     Fm3
......................................................................uucguugucgaugaaUccugccu...................    1       1     Fm3
......................................................................uucguuguGgaugaaaccugccu...................    1       1     Fm3
......................................................................uucguugucgaugCaaccugccu...................    1       1     Fm3
......................................................................uucguugucgaugaaaccugUcu...................    10      1     Fm3
......................................................................uucUuugucgaugaaaccugccu...................    4       1     Fm3
......................................................................uucguugucgUugaaaccugccu...................    30      1     Fm3
......................................................................uucguugucgaugaaaccuUccu...................    9       1     Fm3
......................................................................uuUguugucgaugaaaccugccu...................    13      1     Fm3
......................................................................uucguAgucgaugaaaccugccu...................    2       1     Fm3
......................................................................uucguugucgaugaGaccugccu...................    6       1     Fm3
......................................................................uAcguugucgaugaaaccugccu...................    9       1     Fm3
......................................................................uucguugucgaCgaaaccugccu...................    2       1     Fm3
......................................................................uucAuugucgaugaaaccugccu...................    6       1     Fm3
......................................................................uucguugucgauUaaaccugccu...................    4       1     Fm3
......................................................................uucguugucgaugaaaccAgccu...................    3       1     Fm3
......................................................................uucguugucgaugaaaccugAcu...................    2       1     Fm3
......................................................................uucguugucgauCaaaccugccu...................    2       1     Fm3
......................................................................uucgAugucgaugaaaccugccu...................    4       1     Fm3
......................................................................uucguuAucgaugaaaccugccu...................    7       1     Fm3
......................................................................uuNguugucgaugaaaccugccu...................    1       1     Fm3
......................................................................uucguugucgaugaaaccugccu...................    25599   0     Fm3
......................................................................uucguugucgCugaaaccugccu...................    24      1     Fm3
......................................................................uucguugucgaugaaaGcugccu...................    29      1     Fm3
......................................................................uucguugucgaugaaacUugccu...................    127     1     Fm3
......................................................................Cucguugucgaugaaaccugccu...................    4       1     Fm3
......................................................................uucguugucgaugaaaccugcGu...................    1       1     Fm3
......................................................................uucguugucgaugaaaUcugccu...................    79      1     Fm3
```

Star **Mature**

ccucuauuagaaggaaucaggacauccaaauucaggucucacgacgauucgaacagucauuugaacuuuguucguugucgaugaaaccugccugcugguguccgagucgucga

....uucguugucgaugaaaccugcAu....................	9	1	Fm3
....uucguuCucgaugaaaccugccu....................	4	1	Fm3
....uGcguugucgaugaaaccugccu....................	1	1	Fm3
....uucguugucgaugaaaccugccC....................	44	1	Fm3
....uucguuUucgaugaaaccugccu....................	6	1	Fm3
....uucguugucgaugaaGccugccu....................	1	1	Fm3
....uucguugucCaugaaaccugccu....................	1	1	Fm3
....Aucguugucgaugaaaccugccu....................	4	1	Fm3
....Nucguugucgaugaaaccugccu....................	9	1	Fm3
....uucgGugucgaugaaaccugccu....................	1	1	Fm3
....uucguugucgaugaaaccugccuU...................	49	1	Fm3
....uucguugucgaugaaaccugccugU..................	1	1	Fm3
....uucguugucgaugaaaccugccugUu.................	1	1	Fm3
....ucguugucgaugaaaccug.......................	2	0	Fm3
....ucguugucgaugaaaccugc......................	1	0	Fm3
....ucguugucgaugaaaccugcc.....................	63	0	Fm3
....ucguugucgaugaaaccugUc.....................	1	1	Fm3
....ucguugucgaugGaaccugcc.....................	1	1	Fm3
....ucguugucCaugaaaccugcc.....................	1	1	Fm3
....ucguugucgaAgaaaccugcc.....................	1	1	Fm3
....ucguugucgaugaaaccugcU.....................	3	1	Fm3
....ucguugucgaugaaacAugcc.....................	1	1	Fm3
....ucguugucgGugaaaccugccu....................	1	1	Fm3
....ucguugucgaugaaaccugccA....................	1	1	Fm3
....ucguugucgaAgaaaccugccu....................	2	1	Fm3
....ucguugucgaugaaaccuUccu....................	1	1	Fm3
....ucguugucgaugGaaccugccu....................	1	1	Fm3
....ucguugucgaugaaaccugccu....................	103	0	Fm3
....ucguugucgaugaaaccugcUu....................	1	1	Fm3
....ucUuugucgaugaaaccugccu....................	1	1	Fm3
....ucguCgucgaugaaaccugccu....................	1	1	Fm3
....cguugucgaugaaaccug.......................	6	0	Fm3
....cguugucgaugaaaccuUcc.....................	1	1	Fm3
....cguugucgaugaaaccugcc.....................	10	0	Fm3
....cguugucgUugaaaccugcc.....................	1	1	Fm3
....cguugucgaugaaaccugccu....................	22	0	Fm3
....cguugAcgaugaaaccugccu....................	1	1	Fm3
....guugucgaugaaaccugcc.....................	1	0	Fm3
....uugucgaugaaaccugccu....................	1	0	Fm3
...............................ucaggucucacgacgauucga...	1	0	Mm3
...............................ucaggucucacgacgauucgaac...	2	0	Mm3
...............................ucaggucucacgacgauucgaacU..	3	1	Mm3
................................caggucucacgacgauucga...	4	0	Mm3
................................caggucucacgacgauucgGac...	1	1	Mm3
................................caggucucacgacgauucgaac...	7	0	Mm3
................................caggucucacgacgauucgaacU..	1	1	Mm3
................................caggucucacgacgauucgaacC..	1	1	Mm3
................................caggucucacgacgauucgaaca..	5	0	Mm3
................................caggucucacgacgauucgaacagu..	2	0	Mm3
................................caggucucacgacgauucgaacaguc...	1	0	Mm3
................................caggucucacgacgauucgaacaguca..	1	0	Mm3
...................................gucucacgacgauucgaacagu..	2	0	Mm3
....gucauuugaacuuuguucguugucgaugaaaccugcc.....................	1	0	Mm3
....gucauuugaacuuuguucguugucgaugaaaccugccA....................	1	1	Mm3
....Cuuugaacuuuguucguugucgaugaaacc.........................	1	1	Mm3
....uuugaacuuuguucguugucgaug..............................	1	0	Mm3
....gaacuuuguucguugucgaugaaaccug.......................	1	0	Mm3
....gaacuuuguucguuguAgaugaaaccug.......................	1	1	Mm3
....aacuuuguucguugucgaugaaaccug.......................	1	0	Mm3
....cuuuguucguugucgaugaaaccu........................	1	0	Mm3
....uuuguucguugucgaugaaaccu........................	1	0	Mm3
....guucguugucgaugaaaccu........................	1	0	Mm3
....guucguugucgaugaaaccug.......................	2	0	Mm3
....guucguugucgaugaaaAcug.......................	1	1	Mm3
....guucguugucgaugaaaccugc......................	3	0	Mm3
....Cuucguugucgaugaaaccugc......................	1	1	Mm3
....Uuucguugucgaugaaaccugcc.....................	1	1	Mm3
....guucguugucgaugaaaUcugcc.....................	2	1	Mm3
....guucguugucgaugaaaccugcc.....................	6	0	Mm3

Star **Mature**

```
ccucuauuagaaggaaucaggacauccaaauucaggucucacgacgauucgaacagucauuugaacuuuguucguugucgaugaaaccugccugcugguguccgagucgucga
.....................................................................Cuucguugucgaugaaaccugcc.....................  16     1  Mm3
.....................................................................guucguugucgaugaaaccugcU.....................  1      1  Mm3
.....................................................................guucguugucgaugaaaccugccu....................  9      0  Mm3
.....................................................................Cuucguugucgaugaaaccugccu....................  10     1  Mm3
.....................................................................Uuucguugucgaugaaaccugccu....................  1      1  Mm3
......................................................................uucguugucgaugaaacU.........................  1      1  Mm3
......................................................................uucguugucgaugaaacc.........................  8      0  Mm3
......................................................................uucguugucgaugaaaccu........................  96     0  Mm3
......................................................................uucguugucgaugaaacAu........................  1      1  Mm3
......................................................................uucguugucgaugaaacGu........................  2      1  Mm3
......................................................................uucguugucgaugaaaccG........................  4      1  Mm3
......................................................................uucguugucgaugaaaccGg.......................  1      1  Mm3
......................................................................uucguugucgaugaaaccug.......................  93     0  Mm3
......................................................................uucguugucgaugaaaGcugc......................  1      1  Mm3
......................................................................uucguugucAaugaaaccugc......................  1      1  Mm3
......................................................................uucguugucgaugaaaUcugc......................  2      1  Mm3
......................................................................uucguugucgauAaaaccugc......................  1      1  Mm3
......................................................................uucguugucgGugaaaccugc......................  1      1  Mm3
......................................................................uucguugucgaGgaaaccugc......................  1      1  Mm3
......................................................................uucguugucgaugaaaccugG......................  1      1  Mm3
......................................................................uucguugucgaugUaaccugc......................  1      1  Mm3
......................................................................uucguugucgaugaaacUugc......................  5      1  Mm3
......................................................................uucguugucgaugaaacAugc......................  10     1  Mm3
......................................................................uucgCugucgaugaaaccugc......................  2      1  Mm3
......................................................................uucguugucgUugaaaccugc......................  1      1  Mm3
......................................................................uucguugucgaugaaacGugc......................  3      1  Mm3
......................................................................uucguugucgaugaaaccugA......................  1      1  Mm3
......................................................................uucguugucgaugaUaccugc......................  1      1  Mm3
......................................................................uucguugucgaugaaaccugU......................  32     1  Mm3
......................................................................uucguugucgaugaaaccugc......................  1008   0  Mm3
......................................................................uucguugucUaugaaaccugc......................  1      1  Mm3
......................................................................uucguugucgaugaaaAcugc......................  1      1  Mm3
......................................................................Nucguugucgaugaaaccugc......................  1      1  Mm3
......................................................................uucguugucAaugaaaccugcc.....................  8      1  Mm3
......................................................................Cucguugucgaugaaaccugcc.....................  10     1  Mm3
......................................................................uucguugucgaugaaaccugcA.....................  29     1  Mm3
......................................................................Aucguugucgaugaaaccugcc.....................  3      1  Mm3
......................................................................uAcguugucgaugaaaccugcc.....................  12     1  Mm3
......................................................................uucguugucgauUaaaccugcc.....................  3      1  Mm3
......................................................................uucguuAucgaugaaaccugcc.....................  4      1  Mm3
......................................................................uucguNgucgaugaaaccugcc.....................  4      1  Mm3
......................................................................uucguugucgaugaaacAugcc.....................  197    1  Mm3
......................................................................uuUguugucgaugaaaccugcc.....................  8      1  Mm3
......................................................................uucguAgucgaugaaaccugcc.....................  1      1  Mm3
......................................................................uucguugucgaugaaacUugcc.....................  129    1  Mm3
......................................................................uucguuCucgaugaaaccugcc.....................  2      1  Mm3
......................................................................uucguugucgaGgaaaccugcc.....................  5      1  Mm3
......................................................................uucguugucgaugaaaccugcc.....................  24375  0  Mm3
......................................................................uucguugucgaugaaaccugAc.....................  2      1  Mm3
......................................................................uucUuugucgaugaaaccugcc.....................  2      1  Mm3
......................................................................uCcguugucgaugaaaccugcc.....................  5      1  Mm3
......................................................................uucguugucgauAaaaccugcc.....................  1      1  Mm3
......................................................................uucguugucgaugaGaccugcc.....................  2      1  Mm3
......................................................................uucguugucgCugaaaccugcc.....................  32     1  Mm3
......................................................................uucguCgucgaugaaaccugcc.....................  2      1  Mm3
......................................................................uucguugAcgaugaaaccugcc.....................  2      1  Mm3
......................................................................uucgAugucgaugaaaccugcc.....................  2      1  Mm3
......................................................................uucguGgucgaugaaaccugcc.....................  3      1  Mm3
......................................................................uucguugucgaugaaaccCgcc.....................  3      1  Mm3
......................................................................uucguugucgaugaUaccugcc.....................  2      1  Mm3
......................................................................uucguugucgaugCaaccugcc.....................  1      1  Mm3
......................................................................uucguugucgauCaaaccugcc.....................  2      1  Mm3
......................................................................uuAguugucgaugaaaccugcc.....................  3      1  Mm3
......................................................................Nucguugucgaugaaaccugcc.....................  13     1  Mm3
......................................................................uucguugucgaugaaaccuUcc.....................  8      1  Mm3
......................................................................uucAuugucgaugaaaccugcc.....................  1      1  Mm3
......................................................................uucguugucgaugaaaAcugcc.....................  109    1  Mm3
......................................................................uuGguugucgaugaaaccugcc.....................  3      1  Mm3
......................................................................uucguugucgaugGaaccugcc.....................  2      1  Mm3
......................................................................uucguuUucgaugaaaccugcc.....................  7      1  Mm3
```

Star Mature

ccucuauuagaaggaaucaggacauccaaauucaggucucacgacgauucgaacagucauuugaacuuuguucguugucgaugaaaccugccugcugguguccgagucgucga

....uucguuNucgaugaaaccugcc.....................	4	1	Mm3
....uucguugucgaugaaaccGgcc.....................	1	1	Mm3
....uucguugucgaAgaaaccugcc.....................	3	1	Mm3
....uucguugucgaugaaaccAgcc.....................	1	1	Mm3
....uucguugucgaugaaaccugUc.....................	6	1	Mm3
....uucCuugucgaugaaaccugcc.....................	1	1	Mm3
....uucguugucgaugaCaccugcc.....................	1	1	Mm3
....uucguugucgaugUaaccugcc.....................	3	1	Mm3
....uucguugucgUugaaaccugcc.....................	26	1	Mm3
....uucgCugucgaugaaaccugcc.....................	3	1	Mm3
....uucguugucgaugaaaccuCcc.....................	2	1	Mm3
....uucguugucgaCgaaaccugcc.....................	1	1	Mm3
....uucguugucgaugaaaUcugcc.....................	62	1	Mm3
....uucguugucUaugaaaccugcc.....................	3	1	Mm3
....uucguugucgaugaaaccugcU.....................	275	1	Mm3
....uucguugucCaugaaaccugcc.....................	2	1	Mm3
....uucguugucgaugaaUccugcc.....................	2	1	Mm3
....uucguugucgaugaaaGcugcc.....................	29	1	Mm3
....uucguugucgaugaaaccuAcc.....................	1	1	Mm3
....uucguugCcgaugaaaccugcc.....................	6	1	Mm3
....uucguuguUgaugaaaccugcc.....................	11	1	Mm3
....uucguugucgGugaaaccugcc.....................	28	1	Mm3
....uucguugucgaugaaacGugcc.....................	112	1	Mm3
....uucguugGcgaugaaaccugcc.....................	2	1	Mm3
....uucguugucgCugaaaccugccu....................	35	1	Mm3
....uucguugucgauAaaaccugccu....................	2	1	Mm3
....uucguugucgaugaaaccugccC....................	203	1	Mm3
....uucguugucgaugaaaccAgccu....................	2	1	Mm3
....uucguugucgaugaaacGugccu....................	131	1	Mm3
....Aucguugucgaugaaaccugccu....................	3	1	Mm3
....uucguuUucgaugaaaccugccu....................	4	1	Mm3
....uucguugucgaugGaaccugccu....................	5	1	Mm3
....uucguuguUgaugaaaccugccu....................	13	1	Mm3
....uucguugucgaugCaaccugccu....................	4	1	Mm3
....uucguugucgaugUaaccugccu....................	2	1	Mm3
....uucguugucgaugaaaccugcAu....................	8	1	Mm3
....uucguugucgaugaaaccugGcu....................	3	1	Mm3
....uucguugucgaugaUaccugccu....................	2	1	Mm3
....uucguugucgaugaaaccugccG....................	81	1	Mm3
....uucguugucgauUaaaccugccu....................	1	1	Mm3
....uucguugucgaugaaaccCgccu....................	3	1	Mm3
....uucguugucgaugaCaccugccu....................	1	1	Mm3
....uucguugucgaugaaaccuCccu....................	2	1	Mm3
....uucguugucgaugaaaccugcUu....................	37	1	Mm3
....uucguuAucgaugaaaccugccu....................	5	1	Mm3
....uucguugucgauCaaaccugccu....................	1	1	Mm3
....uucguugucgaAgaaaccugccu....................	1	1	Mm3
....uucguugucgaugaaaccuAccu....................	5	1	Mm3
....uAcguugucgaugaaaccugccu....................	11	1	Mm3
....uucguugCcgaugaaaccugccu....................	2	1	Mm3
....uucguugucgGugaaaccugccu....................	24	1	Mm3
....uuUguugucgaugaaaccugccu....................	8	1	Mm3
....uucguugucgaugaaaccuUccu....................	7	1	Mm3
....uucguugucAaugaaaccugccu....................	7	1	Mm3
....uucguugucgaCgaaaccugccu....................	4	1	Mm3
....uCcguugucgaugaaaccugccu....................	3	1	Mm3
....uucguugucgaugaaaccugccu....................	26528	0	Mm3
....uuGguugucgaugaaaccugccu....................	10	1	Mm3
....uucgGugucgaugaaaccugccu....................	1	1	Mm3
....uucguugucgaugaaaUcugccu....................	83	1	Mm3
....uucguugucgaugaaGccugccu....................	3	1	Mm3
....uucAuugucgaugaaaccugccu....................	6	1	Mm3
....uucUuugucgaugaaaccugccu....................	1	1	Mm3
....uucguugucgaugaaCccugccu....................	2	1	Mm3
....uucguugucCaugaaaccugccu....................	1	1	Mm3
....uGcguugucgaugaaaccugccu....................	5	1	Mm3
....uucguugucgaugaaaccugAcu....................	5	1	Mm3
....uucguugucgaugaaaccugUcu....................	9	1	Mm3
....uucguugucgUugaaaccugccu....................	33	1	Mm3
....uucguugucgaugaaacUugccu....................	152	1	Mm3

```
                               Star                          Mature
ccucuauuagaaggaaucaggacauccaaauucaggucucacgacgauucgaacagucauuugaacuuuguucguugucgaugaaaccugccugcugguguccgagucgucga
......................................................................uucgCugucgaugaaaccugccu....................    6     1    Mm3
......................................................................Nucguugucgaugaaaccugccu....................    9     1    Mm3
......................................................................uucguAgucgaugaaaccugccu....................    2     1    Mm3
......................................................................uucguugucgaugaaaccugccA....................    377   1    Mm3
......................................................................uucguugucgaugaGaccugccu....................    6     1    Mm3
......................................................................uucguNgucgaugaaaccugccu....................    5     1    Mm3
......................................................................Cucguugucgaugaaaccugccu....................    3     1    Mm3
......................................................................uucguugucgaGgaaaccugccu....................    1     1    Mm3
......................................................................uucguugucgaugaaaccGgccu....................    1     1    Mm3
......................................................................uucgAugucgaugaaaccugccu....................    4     1    Mm3
......................................................................uNcguugucgaugaaaccugccu....................    1     1    Mm3
......................................................................uucguugucgaugaaUccugccu....................    3     1    Mm3
......................................................................uucguuNucgaugaaaccugccu....................    2     1    Mm3
......................................................................uucguugucgaugaaacAugccu....................    233   1    Mm3
......................................................................uucguugucgaugaaaGcugccu....................    26    1    Mm3
......................................................................uuAguugucgaugaaaccugccu....................    9     1    Mm3
......................................................................uucguuguAgaugaaaccugccu....................    2     1    Mm3
......................................................................uucguugAcgaugaaaccugccu....................    3     1    Mm3
......................................................................uucguCgucgaugaaaccugccu....................    2     1    Mm3
......................................................................uucguGgucgaugaaaccugccu....................    4     1    Mm3
......................................................................uucguugucgaugaaaAcugccu....................    93    1    Mm3
......................................................................uucguugucgaugaaaccCgccug...................    1     1    Mm3
......................................................................uucguugucgaugaaaccugccuU...................    93    1    Mm3
......................................................................uucguugucgaugaaaccugccuUcu.................    2     1    Mm3
.......................................................................ucguugucgaugaaaccu........................    2     0    Mm3
.......................................................................ucguugAcgaugaaaccugcc.....................    1     1    Mm3
.......................................................................ucguugGcgaugaaaccugcc.....................    1     1    Mm3
.......................................................................ucguugucgaugaaaccugcc.....................    63    0    Mm3
.......................................................................ucCuugucgaugaaaccugcc.....................    1     1    Mm3
.......................................................................ucguugucgaugaaaAcugcc.....................    1     1    Mm3
.......................................................................ucguugucgaCgaaaccugcc.....................    1     1    Mm3
.......................................................................ucguugucCaugaaaccugcc.....................    1     1    Mm3
.......................................................................ucgAugucgaugaaaccugcc.....................    1     1    Mm3
.......................................................................ucguugucgaugaaaccugccu....................    82    0    Mm3
.......................................................................ucguugucgaGgaaaccugccu....................    1     1    Mm3
.......................................................................uUguugucgaugaaaccugccu....................    2     1    Mm3
.......................................................................ucUuugucgaugaaaccugccu....................    1     1    Mm3
.......................................................................ucguugucgaugaaaccugcUu....................    1     1    Mm3
........................................................................cguugucgaugaaaccugc......................    3     0    Mm3
........................................................................cguugucgaugaaaccugcc.....................    2     0    Mm3
........................................................................cguugucgaugaaaccuUccu....................    1     1    Mm3
........................................................................cguugucgaugaaaccugccu....................    8     0    Mm3
.........................................................................guugucgaugaaaccugcc.....................    1     0    Mm3
.........................................................................guugucgaugaaaccugccu....................    1     0    Mm3
.........................................................................guugucgaugGaaccugccu....................    1     1    Mm3

................................caggucucacgacgauuUga.............................................................    1     1    Fm1
................................caggucucacgacgauucga.............................................................    6     0    Fm1
................................caggucucacgacgauucgaaca..........................................................    4     0    Fm1
.............................................gauucgaacagucauuugaacuuuguucguugucgaugaaaccug.......................    1     0    Fm1
...................................................aacagucauuugaacuuuguucguugucgaugaaaccugU......................    1     1    Fm1
...........................................................uuugaacuuuguucguugucgau...............................    3     0    Fm1
..............................................................gaacuuuguucguugucgaugaaaccug.......................    1     0    Fm1
.................................................................cuuuguucguugucgaugaaaccGg.......................    1     1    Fm1
.................................................................cuuuguucguugucgaugaaacAug.......................    1     1    Fm1
.................................................................cuuuguucguugucgaugaaaccug.......................    8     0    Fm1
...................................................................uuguucguugucgaugaaaccug.......................    3     0    Fm1
....................................................................Gguucguugucgaugaaaccugcc.....................    3     1    Fm1
.....................................................................guucguugucgaugaaaccug.......................    1     0    Fm1
.....................................................................guucguugucgaugaaaccugcc.....................    2     0    Fm1
.....................................................................guucguugucgaugaaaccugcU.....................    4     1    Fm1
.....................................................................Cuucguugucgaugaaaccugccu....................    3     1    Fm1
.....................................................................guucguugucgaugaCaccugccu....................    1     1    Fm1
.....................................................................guucguugucgaugaaaccugccu....................    6     0    Fm1
......................................................................uucguugucgaugaaacc.........................    2     0    Fm1
......................................................................uucguugucgaugaaaccG........................    4     1    Fm1
......................................................................uucguugucgaugaaaccu........................    29    0    Fm1
......................................................................uucguugucgaugaaaAcug.......................    2     1    Fm1
......................................................................uucguugucgCugaaaccug.......................    2     1    Fm1
......................................................................uucguugucgaugaaacGug.......................    1     1    Fm1
```

Star   Mature

```
ccucuauuagaaggaaucaggacauccaaauucaggucucacgacgauucgaacagucauuugaacuuuguucguugucgaugaaaccugccugcugguguccgagucgucga
......................................................................uuGguugucgaugaaaccug.......................   1     1    Fm1
......................................................................uucguugucgaugaaacUug.......................   3     1    Fm1
......................................................................uucgGugucgaugaaaccug.......................   1     1    Fm1
......................................................................uucguugucgaugaaaccug.......................   678   0    Fm1
......................................................................uucguugucgaugaaaccGg.......................   10    1    Fm1
......................................................................uucguugucgaugaaacAug.......................   7     1    Fm1
......................................................................uuUguugucgaugaaaccug.......................   1     1    Fm1
......................................................................uucguugucgaugaaaUcug.......................   2     1    Fm1
......................................................................uucguugucgUugaaaccug.......................   1     1    Fm1
......................................................................uucguugucgaugUaaccug.......................   1     1    Fm1
......................................................................uucguugucgaugaaaccuA.......................   3     1    Fm1
......................................................................uucguuguGgaugaaaccug.......................   1     1    Fm1
......................................................................uucguugucgaugaaaGcug.......................   1     1    Fm1
......................................................................uAcguugucgaugaaaccug.......................   1     1    Fm1
......................................................................uucguugucgaugaaaccugc......................   982   0    Fm1
......................................................................uucguuguAgaugaaaccugc......................   1     1    Fm1
......................................................................uucguugucgaCgaaaccugc......................   1     1    Fm1
......................................................................uucguugucgaugaaacGugc......................   4     1    Fm1
......................................................................uuGguugucgaugaaaccugc......................   1     1    Fm1
......................................................................uucguugucgaugaaacUugc......................   6     1    Fm1
......................................................................uGcguugucgaugaaaccugc......................   2     1    Fm1
......................................................................uuAguugucgaugaaaccugc......................   1     1    Fm1
......................................................................uucguugucgaugaaaccugG......................   5     1    Fm1
......................................................................uucguugAcgaugaaaccugc......................   1     1    Fm1
......................................................................uucguugucgaugaaaGcugc......................   1     1    Fm1
......................................................................uucguugucgaugaaaAcugc......................   3     1    Fm1
......................................................................uucguugucgaugaaaccugU......................   43    1    Fm1
......................................................................uucguugucgaugaaacAugc......................   9     1    Fm1
......................................................................uucguCgucgaugaaaccugc......................   1     1    Fm1
......................................................................uucguugucgaugaaaUcugc......................   2     1    Fm1
......................................................................uuAguugucgaugaaaccugcc.....................   9     1    Fm1
......................................................................uucguugucgaugaaaAcugcc.....................   119   1    Fm1
......................................................................uucguugucgaugaaaccuCcc.....................   8     1    Fm1
......................................................................uucAuugucgaugaaaccugcc.....................   1     1    Fm1
......................................................................uucguugucgaugGaaccugcc.....................   7     1    Fm1
......................................................................Gucguugucgaugaaaccugcc.....................   2     1    Fm1
......................................................................uucguugucgCugaaaccugcc.....................   42    1    Fm1
......................................................................uucguugucgaugaaUccugcc.....................   6     1    Fm1
......................................................................uucguugucgaugaaacAugcc.....................   292   1    Fm1
......................................................................uucguugucgaugaaaccCgcc.....................   5     1    Fm1
......................................................................uucguuNucgaugaaaccugcc.....................   2     1    Fm1
......................................................................uucguugucgaugaaCccugcc.....................   1     1    Fm1
......................................................................uAcguugucgaugaaaccugcc.....................   12    1    Fm1
......................................................................uucguugAcgaugaaaccugcc.....................   1     1    Fm1
......................................................................uucguugucgaugaaaccAgcc.....................   1     1    Fm1
......................................................................uucgCugucgaugaaaccugcc.....................   5     1    Fm1
......................................................................uuUguugucgaugaaaccugcc.....................   21    1    Fm1
......................................................................uucgAugucgaugaaaccugcc.....................   5     1    Fm1
......................................................................uucguGgucgaugaaaccugcc.....................   4     1    Fm1
......................................................................uucguugucgaugaaaccuAcc.....................   7     1    Fm1
......................................................................uucguugucgaugaaaccugAc.....................   2     1    Fm1
......................................................................uucguugucCaugaaaccugcc.....................   2     1    Fm1
......................................................................uucguuguAgaugaaaccugcc.....................   1     1    Fm1
......................................................................Cucguugucgaugaaaccugcc.....................   9     1    Fm1
......................................................................uucguugucgaugCaaccugcc.....................   1     1    Fm1
......................................................................uucUuugucgaugaaaccugcc.....................   1     1    Fm1
......................................................................uucguugucgaAgaaaccugcc.....................   1     1    Fm1
......................................................................uucguugucgaGgaaaccugcc.....................   4     1    Fm1
......................................................................uucguugucgaugUaaccugcc.....................   1     1    Fm1
......................................................................uucguCgucgaugaaaccugcc.....................   5     1    Fm1
......................................................................uCcguugucgaugaaaccugcc.....................   2     1    Fm1
......................................................................uucguugucgaugaaaccugcU.....................   510   1    Fm1
......................................................................uucguugucgaugaaGccugcc.....................   3     1    Fm1
......................................................................uucguugGcgaugaaaccugcc.....................   1     1    Fm1
......................................................................uucguNgucgaugaaaccugcc.....................   3     1    Fm1
......................................................................uucguuCucgaugaaaccugcc.....................   1     1    Fm1
......................................................................uucguugucgaugaGaccugcc.....................   5     1    Fm1
......................................................................uucguugucUaugaaaccugcc.....................   6     1    Fm1
......................................................................Aucguugucgaugaaaccugcc.....................   3     1    Fm1
......................................................................uucguugucgaugaaaccugcA.....................   42    1    Fm1
```

Star Mature

ccucuauuagaaggaaucaggacauccaaauucaggucucacgacgauucgaacagucauuugaacuuuguucguugucgaugaaaccugccugcugguguccgagucgucga			
....uucguugucgaugaCaccugcc.....................	2	1	Fm1
....uucguuguGgaugaaaccugcc.....................	2	1	Fm1
....uucguugucgaugaaaccGgcc.....................	1	1	Fm1
....uGcguugucgaugaaaccugcc.....................	2	1	Fm1
....uucguugucAaugaaaccugcc.....................	7	1	Fm1
....uucguugucgaCgaaaccugcc.....................	6	1	Fm1
....uucguugCcgaugaaaccugcc.....................	1	1	Fm1
....uucguugucgaugaaaccugUc.....................	7	1	Fm1
....uucguugucgaugaaaccugcG.....................	8	1	Fm1
....uucguugucgauCaaaccugcc.....................	4	1	Fm1
....uucguugucgaugaaaccugcc.....................	31362	0	Fm1
....uucguugucgaugaaaccugGc.....................	1	1	Fm1
....uucCuugucgaugaaaccugcc.....................	4	1	Fm1
....uucguugucgGugaaaccugcc.....................	43	1	Fm1
....uucguugucgaugaaaccuUcc.....................	14	1	Fm1
....uucguuAucgaugaaaccugcc.....................	11	1	Fm1
....uucguugucgaugaaaUcugcc.....................	70	1	Fm1
....uucguugucgauUaaaccugcc.....................	2	1	Fm1
....uucguugucgaugaaacUugcc.....................	148	1	Fm1
....uucguuguUgaugaaaccugcc.....................	8	1	Fm1
....uuGguugucgaugaaaccugcc.....................	8	1	Fm1
....uucguAgucgaugaaaccugcc.....................	3	1	Fm1
....uucguugucgUugaaaccugcc.....................	40	1	Fm1
....uucguugucgaugaaacGugcc.....................	122	1	Fm1
....uucguuUucgaugaaaccugcc.....................	7	1	Fm1
....uucguugucgaugaUaccugcc.....................	1	1	Fm1
....Nucguugucgaugaaaccugcc.....................	12	1	Fm1
....uucguugucgaugaaaGcugcc.....................	58	1	Fm1
....uucguugucgauAaaaccugcc.....................	3	1	Fm1
....uucguugucgaugaaGccugccu....................	4	1	Fm1
....uucguugucgaugCaaccugccu....................	3	1	Fm1
....uucguGgucgaugaaaccugccu....................	5	1	Fm1
....uucguugucgaugaaaccuCccu....................	4	1	Fm1
....uuGguugucgaugaaaccugccu....................	3	1	Fm1
....uucguugucgaugaaaccGgccu....................	1	1	Fm1
....uucguugucgaAgaaaccugccu....................	4	1	Fm1
....uucguugucUaugaaaccugccu....................	3	1	Fm1
....uGcguugucgaugaaaccugccu....................	2	1	Fm1
....uucguugucgauUaaaccugccu....................	3	1	Fm1
....uucguugucgaugaaaccugccA....................	1020	1	Fm1
....uucguuNucgaugaaaccugccu....................	1	1	Fm1
....uucguugucgaugaaCccugccu....................	1	1	Fm1
....uucguugucgGugaaaccugccu....................	40	1	Fm1
....uucguugucgaugaaaccugGcu....................	2	1	Fm1
....uucguugucgaugaaacGugccu....................	148	1	Fm1
....Aucguugucgaugaaaccugccu....................	4	1	Fm1
....uucguugucgaugGaaccugccu....................	2	1	Fm1
....uuUguugucgaugaaaccugccu....................	19	1	Fm1
....uucguNgucgaugaaaccugccu....................	5	1	Fm1
....uucguugucgaugaaaccugcUu....................	29	1	Fm1
....uucguuguAgaugaaaccugccu....................	3	1	Fm1
....uucguugucgaugUaaccugccu....................	4	1	Fm1
....uucgCugucgaugaaaccugccu....................	6	1	Fm1
....uucguugucgauCaaaccugccu....................	3	1	Fm1
....uucguugAcgaugaaaccugccu....................	3	1	Fm1
....uucguugucgaugaaUccugccu....................	4	1	Fm1
....uAcguugucgaugaaaccugccu....................	7	1	Fm1
....uucguugucgaGgaaaccugccu....................	2	1	Fm1
....uucguugucgaugaaacUugccu....................	202	1	Fm1
....uucguugucgaugaaaAcugccu....................	141	1	Fm1
....uucguugCcgaugaaaccugccu....................	5	1	Fm1
....uucgAugucgaugaaaccugccu....................	2	1	Fm1
....uucguuAucgaugaaaccugccu....................	4	1	Fm1
....uucguuguGgaugaaaccugccu....................	2	1	Fm1
....uucCuugucgaugaaaccugccu....................	1	1	Fm1
....uuAguugucgaugaaaccugccu....................	9	1	Fm1
....uucguugGcgaugaaaccugccu....................	1	1	Fm1
....uucguugucgaugaaaccugcAu....................	30	1	Fm1
....uucguuUucgaugaaaccugccu....................	4	1	Fm1
....uucguugucgaugaaacAugccu....................	286	1	Fm1

Star Mature

```
ccucuauuagaaggaaucaggacauccaaauucaggucucacgacgauucgaacagucauuugaacuuuguucguugucgaugaaaccugccugcugguguccgagucgucga
......................................................................uucguugucgaugaaaccAgccu....................     4      1    Fm1
......................................................................uucAuugucgaugaaaccugccu....................     10     1    Fm1
......................................................................uucguugucgauAaaaccugccu....................     5      1    Fm1
......................................................................uucguAgucgaugaaaccugccu....................     2      1    Fm1
......................................................................uucguugucgaugaGaccugccu....................     6      1    Fm1
......................................................................uucguugucgaugaaaUcugccu....................     76     1    Fm1
......................................................................uCcguugucgaugaaaccugccu....................     3      1    Fm1
......................................................................uucguugucgaugaaaccugccu....................     33105  0    Fm1
......................................................................uucguugucgaugaaaccugAcu....................     6      1    Fm1
......................................................................uucguugucgaugaaaGcugccu....................     45     1    Fm1
......................................................................uucguugucgaugaaaccuUccu....................     12     1    Fm1
......................................................................uucguugucgaugaaaccCgccu....................     1      1    Fm1
......................................................................uucguugucgaugaaaccuAccu....................     4      1    Fm1
......................................................................uucgGugucgaugaaaccugccu....................     4      1    Fm1
......................................................................uucguugucgaugaaaccugccG....................     171    1    Fm1
......................................................................uucguuCucgaugaaaccugccu....................     3      1    Fm1
......................................................................uucUuugucgaugaaaccugccu....................     3      1    Fm1
......................................................................Cucguugucgaugaaaccugccu....................     4      1    Fm1
......................................................................uNcguugucgaugaaaccugccu....................     2      1    Fm1
......................................................................uucguugucgaugaaaccugcGu....................     1      1    Fm1
......................................................................uucguugucgaugaaaccugccC....................     220    1    Fm1
......................................................................uucguugucgaCgaaaccugccu....................     4      1    Fm1
......................................................................uucguugucgCugaaaccugccu....................     37     1    Fm1
......................................................................uucguugucgaugaaaccugUcu....................     9      1    Fm1
......................................................................uucguuguUgaugaaaccugccu....................     25     1    Fm1
......................................................................Nucguugucgaugaaaccugccu....................     17     1    Fm1
......................................................................uucguugucgUugaaaccugccu....................     34     1    Fm1
......................................................................uucguugucAaugaaaccugccu....................     4      1    Fm1
......................................................................uucguugucgaugaaaccugccuA...................     3      1    Fm1
......................................................................uucguugucgaugaaaccugccuC...................     7      1    Fm1
......................................................................uucguugucgaugaaaccugccuU...................     82     1    Fm1
......................................................................uucguugucgaugaaaccugccuUcu.................     4      1    Fm1
.......................................................................ucguugucgaugaaaccug.......................     3      0    Fm1
.......................................................................ucguugucgaugaaaccugc......................     2      0    Fm1
.......................................................................ucguuguAgaugaaaccugcc.....................     1      1    Fm1
.......................................................................ucguugucgaugaaaccugcU.....................     2      1    Fm1
.......................................................................ucguugucgaCgaaaccugcc.....................     1      1    Fm1
.......................................................................ucguugucgaugaaaccAgcc.....................     1      1    Fm1
.......................................................................ucguugucgaAgaaaccugcc.....................     1      1    Fm1
.......................................................................ucguugAcgaugaaaccugcc.....................     1      1    Fm1
.......................................................................ucguugucgauCaaaccugcc.....................     1      1    Fm1
.......................................................................ucguugucgauUaaaccugcc.....................     1      1    Fm1
.......................................................................ucguugucgaugaaaccugcc.....................     59     0    Fm1
.......................................................................ucguugucgCugaaaccugcc.....................     1      1    Fm1
.......................................................................ucguugucgauUaaaccugccu....................     1      1    Fm1
.......................................................................uUguugucgaugaaaccugccu....................     1      1    Fm1
.......................................................................ucguugucgUugaaaccugccu....................     1      1    Fm1
.......................................................................ucguugucgaugaaaccugccA....................     4      1    Fm1
.......................................................................ucguugucgauAaaaccugccu....................     1      1    Fm1
.......................................................................ucguugucgaugaaacAugccu....................     1      1    Fm1
.......................................................................ucguugucgCugaaaccugccu....................     1      1    Fm1
.......................................................................ucguugucgaugaaaccugccG....................     1      1    Fm1
.......................................................................ucguugucgaCgaaaccugccu....................     1      1    Fm1
.......................................................................ucguugucgaugaaaccugccu....................     95     0    Fm1
........................................................................cguugucgaugaaaccCg.......................     1      1    Fm1
........................................................................cguugucgaugaaaccug.......................     6      0    Fm1
........................................................................cguugucgaugaaaccGg.......................     1      1    Fm1
........................................................................cguugNcgaugaaaccugcc.....................     1      1    Fm1
........................................................................cguugucgaugaaaccugcc.....................     5      0    Fm1
........................................................................cguugucgaugaaaccAgccu....................     1      1    Fm1
........................................................................cguugucgGugaaaccugccu....................     1      1    Fm1
........................................................................cguugucgaugaaaccugccu....................     26     0    Fm1
.........................................................................guugucgaugaaaccugccu....................     3      0    Fm1

....................................................................uguucguugucgaugaaaccugcc.....................     11     0    Fm2
.....................................................................Cuucguugucgaugaaaccugcc.....................     1      1    Fm2
.....................................................................Cuucguugucgaugaaaccugccu....................     4      1    Fm2
......................................................................uucguugucgaugaaacc.........................     1      0    Fm2
......................................................................uucguugucgaugaaaccC........................     1      1    Fm2
......................................................................uucguugucgaugaaaccu........................     8      0    Fm2
```

Star Mature

```
ccucuauuagaaggaaucaggacauccaaauucaggucucacgacgauucgaacagucauuugaacuuuguucguugucgaugaaaccugccugcugguguccgagucguga
......................................................................uucguugucgaugaaacAu.......................   1      1   Fm2
......................................................................uucguugucgaugaaaccug......................   59     0   Fm2
......................................................................uucguugucgaugaaacUug......................   1      1   Fm2
......................................................................uucguugucgaugaaaccGg......................   1      1   Fm2
......................................................................uucguugucgaugaaacGug......................   1      1   Fm2
......................................................................uucguugucgaugaaacAugc.....................   1      1   Fm2
......................................................................uucguugucgaugaaaccugc.....................   369    0   Fm2
......................................................................uucguugucgaugaaacGugc.....................   1      1   Fm2
......................................................................uucguugucgaugaaacUugc.....................   1      1   Fm2
......................................................................uucAuugucgaugaaaccugc.....................   1      1   Fm2
......................................................................uucguugucUaugaaaccugc.....................   1      1   Fm2
......................................................................uucguugucgGugaaaccugc.....................   2      1   Fm2
......................................................................uucguugucgaugaaaccugU.....................   19     1   Fm2
......................................................................uucguugucgaugaUaccugc.....................   1      1   Fm2
......................................................................uucguuAucgaugaaaccugc.....................   1      1   Fm2
......................................................................uucguuUucgaugaaaccugc.....................   2      1   Fm2
......................................................................uucguugucgCugaaaccugc.....................   2      1   Fm2
......................................................................uucguugucgaugaaaUcugc.....................   1      1   Fm2
......................................................................uucguGgucgaugaaaccugc.....................   1      1   Fm2
......................................................................uuUguugucgaugaaaccugc.....................   1      1   Fm2
......................................................................uucguugucgaugaaaAcugc.....................   1      1   Fm2
......................................................................uucguugucgaugaaaGcugc.....................   2      1   Fm2
......................................................................uucguugucgaugaaCccugcc....................   1      1   Fm2
......................................................................uucgAugucgaugaaaccugcc....................   1      1   Fm2
......................................................................uuUguugucgaugaaaccugcc....................   4      1   Fm2
......................................................................uucguuUucgaugaaaccugcc....................   6      1   Fm2
......................................................................uAcguugucgaugaaaccugcc....................   1      1   Fm2
......................................................................uucguugucgaugaaaccugcc....................   12944  0   Fm2
......................................................................uucguugucgaugaaacAugcc....................   98     1   Fm2
......................................................................uucguugucgaugaaacUugcc....................   60     1   Fm2
......................................................................uuGguugucgaugaaaccugcc....................   4      1   Fm2
......................................................................uucguugCcgaugaaaccugcc....................   1      1   Fm2
......................................................................uuAguugucgaugaaaccugcc....................   2      1   Fm2
......................................................................uucguuguAgaugaaaccugcc....................   3      1   Fm2
......................................................................uucguugucgaugaaUccugcc....................   1      1   Fm2
......................................................................uucguugucgaugaaaAcugcc....................   48     1   Fm2
......................................................................uucguugucgCugaaaccugcc....................   24     1   Fm2
......................................................................uucguugucgaugaaacGugcc....................   60     1   Fm2
......................................................................uucguugucgaugaaaccugUc....................   1      1   Fm2
......................................................................uucguuAucgaugaaaccugcc....................   3      1   Fm2
......................................................................uucguugucgaugaCaccugcc....................   2      1   Fm2
......................................................................uucguugucgUugaaaccugcc....................   20     1   Fm2
......................................................................uucguugucgaGgaaaccugcc....................   1      1   Fm2
......................................................................uucguugucgaugaaaccugcA....................   19     1   Fm2
......................................................................uucguGgucgaugaaaccugcc....................   5      1   Fm2
......................................................................uucguugucgaugaaaGcugcc....................   22     1   Fm2
......................................................................uucguugucgaugaaaccugAc....................   2      1   Fm2
......................................................................uucguugucAaugaaaccugcc....................   5      1   Fm2
......................................................................uucguugucgaugaaaccuAcc....................   5      1   Fm2
......................................................................uucCuugucgaugaaaccugcc....................   1      1   Fm2
......................................................................uucguugucUaugaaaccugcc....................   4      1   Fm2
......................................................................uucguugucgaugaaaccugcG....................   1      1   Fm2
......................................................................uucguugucgGugaaaccugcc....................   20     1   Fm2
......................................................................uucguugAcgaugaaaccugcc....................   4      1   Fm2
......................................................................uucguugucgaCgaaaccugcc....................   1      1   Fm2
......................................................................uucguuguGgaugaaaccugcc....................   2      1   Fm2
......................................................................uCcguugucgaugaaaccugcc....................   3      1   Fm2
......................................................................uucguugucgaugaaaccugcU....................   126    1   Fm2
......................................................................Aucguugucgaugaaaccugcc....................   1      1   Fm2
......................................................................uucguugucgaugaaaccGgcc....................   2      1   Fm2
......................................................................Cucguugucgaugaaaccugcc....................   1      1   Fm2
......................................................................uucguugucgaugCaaccugcc....................   1      1   Fm2
......................................................................uucguAgucgaugaaaccugcc....................   1      1   Fm2
......................................................................uucguugucgauCaaaccugcc....................   2      1   Fm2
......................................................................Gucguugucgaugaaaccugcc....................   1      1   Fm2
......................................................................uucguugucgaugaaaccuUcc....................   10     1   Fm2
......................................................................uucguugucgaugaaaccAgcc....................   1      1   Fm2
......................................................................uucguugucgaugaaaccuCcc....................   8      1   Fm2
......................................................................uucAuugucgaugaaaccugcc....................   3      1   Fm2
......................................................................uucguugucgaugaaaccCgcc....................   3      1   Fm2
```

Star Mature

```
ccucuauuagaaggaaucaggacauccaaauucaggucucacgacgauucgaacagucauuugaacuuuguucguugucgaugaaaccugccugcuggugucgagucgucga
......................................................................uucguugucgaAgaaaccugcc....................   1       1   Fm2
......................................................................uucguugucgaugaaGccugcc....................   1       1   Fm2
......................................................................uucguugucgaugaaaUcugcc....................   28      1   Fm2
......................................................................uucguugucgaugaaaccugGc....................   2       1   Fm2
......................................................................uucguNgucgaugaaaccugcc....................   3       1   Fm2
......................................................................uucguugucgauAaaaccugcc....................   1       1   Fm2
......................................................................uucguuguUgaugaaaccugcc....................   8       1   Fm2
......................................................................Nucguugucgaugaaaccugcc....................   2       1   Fm2
......................................................................uucUuugucgaugaaaccugcc....................   3       1   Fm2
......................................................................uucguugGcgaugaaaccugcc....................   4       1   Fm2
......................................................................uucguugucCaugaaaccugcc....................   2       1   Fm2
......................................................................uucguugucgaugGaaccugcc....................   3       1   Fm2
......................................................................uucguugucgaugUaaccugcc....................   1       1   Fm2
......................................................................uucguugucgaugaGaccugcc....................   3       1   Fm2
......................................................................uucguugucgaugaaaccugccC...................   38      1   Fm2
......................................................................uucgCugucgaugaaaccugccu...................   1       1   Fm2
......................................................................uucguuguAgaugaaaccugccu...................   2       1   Fm2
......................................................................uucguugucgaugaaaccugccu...................   11845   0   Fm2
......................................................................uucguugucgaugaaaUcugccu...................   43      1   Fm2
......................................................................uucguugucgaCgaaaccugccu...................   1       1   Fm2
......................................................................uucguugucgaAgaaaccugccu...................   1       1   Fm2
......................................................................uucguugucgaugaaacAugccu...................   101     1   Fm2
......................................................................Nucguugucgaugaaaccugccu...................   11      1   Fm2
......................................................................uucguugucgaugaaaccugcGu...................   3       1   Fm2
......................................................................uucguugucgaugaaaccuUccu...................   10      1   Fm2
......................................................................uucguugucgaugaaGccugccu...................   1       1   Fm2
......................................................................uucguugucgauAaaaccugccu...................   2       1   Fm2
......................................................................uucguuCucgaugaaaccugccu...................   2       1   Fm2
......................................................................uucguugucgaugaUaccugccu...................   1       1   Fm2
......................................................................uucguugAcgaugaaaccugccu...................   2       1   Fm2
......................................................................Cucguugucgaugaaaccugccu...................   1       1   Fm2
......................................................................uucguugGcgaugaaaccugccu...................   1       1   Fm2
......................................................................uuUguugucgaugaaaccugccu...................   11      1   Fm2
......................................................................uucguugucgaugaaaccugcUu...................   14      1   Fm2
......................................................................uuGguugucgaugaaaccugccu...................   2       1   Fm2
......................................................................uucguugucgCugaaaccugccu...................   19      1   Fm2
......................................................................uucguugucgaugGaaccugccu...................   1       1   Fm2
......................................................................uucguugucgUugaaaccugccu...................   20      1   Fm2
......................................................................uucguugucgaugaaaccugccA...................   119     1   Fm2
......................................................................Aucguugucgaugaaaccugccu...................   2       1   Fm2
......................................................................uucguugucgauCaaaccugccu...................   1       1   Fm2
......................................................................uucguugucgaugaaaGcugccu...................   17      1   Fm2
......................................................................uucguugucgaugaaaccugUcu...................   3       1   Fm2
......................................................................uucguugucgaugaaaccCgccu...................   1       1   Fm2
......................................................................uucguAgucgaugaaaccugccu...................   2       1   Fm2
......................................................................uucguNgucgaugaaaccugccu...................   1       1   Fm2
......................................................................uucgGugucgaugaaaccugccu...................   1       1   Fm2
......................................................................uucguugucgaugaaaccugAcu...................   1       1   Fm2
......................................................................uAcguugucgaugaaaccugccu...................   6       1   Fm2
......................................................................uucguugucgaugaaaccAgccu...................   1       1   Fm2
......................................................................uucguugucgaugUaaccugccu...................   2       1   Fm2
......................................................................uucguugucUaugaaaccugccu...................   2       1   Fm2
......................................................................uucguugucCaugaaaccugccu...................   2       1   Fm2
......................................................................uucguugucgaugaaaccuCccu...................   1       1   Fm2
......................................................................uucUuugucgaugaaaccugccu...................   2       1   Fm2
......................................................................uucguugucgaugaaaAcugccu...................   46      1   Fm2
......................................................................uucguugucgaugaCaccugccu...................   1       1   Fm2
......................................................................uucguuUucgaugaaaccugccu...................   2       1   Fm2
......................................................................uucguugucgaugaaaccugcAu...................   3       1   Fm2
......................................................................uucguugucgGugaaaccugccu...................   28      1   Fm2
......................................................................uucguugucAaugaaaccugccu...................   3       1   Fm2
......................................................................uucAuugucgaugaaaccugccu...................   4       1   Fm2
......................................................................uucCuugucgaugaaaccugccu...................   2       1   Fm2
......................................................................uucguuAucgaugaaaccugccu...................   4       1   Fm2
......................................................................uucguugCcgaugaaaccugccu...................   2       1   Fm2
......................................................................uucguugucgaugaaaccugccG...................   100     1   Fm2
......................................................................uucguugucgaugaaacUugccu...................   95      1   Fm2
......................................................................uucguugucgaugaaaccuAccu...................   5       1   Fm2
......................................................................uucguugucgaugaaacGugccu...................   57      1   Fm2
......................................................................uucguCgucgaugaaaccugccu...................   1       1   Fm2
```

```
                               Star                                 Mature
ccucuauuagaaggaaucaggacauccaaauucaggucucacgacgauucgaacagucauuugaacuuuguucguugucgaugaaaccugccugcuggugucgagucguga
......................................................................uucguugucgaugaGaccugccu..................    2     1    Fm2
......................................................................uuAguugucgaugaaaccugccu..................    1     1    Fm2
......................................................................uucguuguUgaugaaaccugccu..................    4     1    Fm2
......................................................................uucguugucgaugaaaccugccuU.................    7     1    Fm2
.......................................................................ucguugucgaugaaaccugcc...................    42    0    Fm2
.......................................................................ucguugucgUugaaaccugcc...................    1     1    Fm2
.......................................................................ucguugucgaugaaaccugcU...................    3     1    Fm2
.......................................................................ucguugucgaugaaaccugccu..................    48    0    Fm2
.......................................................................ucguugucgaugaaaccugccG..................    1     1    Fm2
........................................................................cguugucgaugaaaccugcc...................    1     0    Fm2
........................................................................cguugucgaugaaaccugccu..................    3     0    Fm2
..........................................................................uugucgaugaaaccugccu..................    8     0    Fm2

.....................................................................Cuucguugucgaugaaaccugccu..................    1     1    Mm1
.....................................................................guucguugucgaugaaaccugccu..................    10    0    Mm1
......................................................................uucguugucgaugaaacc.......................    8     0    Mm1
......................................................................uucguugucgUugaaaccu......................    1     1    Mm1
......................................................................uucguugucgaugaaaccu......................    49    0    Mm1
......................................................................uucguugucgaugaaacUu......................    1     1    Mm1
......................................................................uucguugucgaugaaaGcu......................    1     1    Mm1
......................................................................uucguugucgaugaaacAu......................    1     1    Mm1
......................................................................uucguugucgGugaaaccu......................    1     1    Mm1
......................................................................uucguugucgaugaaaUcug.....................    1     1    Mm1
......................................................................uucguugucgaugGaaccug.....................    1     1    Mm1
......................................................................uucguugucgaugaaaccug.....................    56    0    Mm1
......................................................................uucguugucgaugaaaAcugc....................    1     1    Mm1
......................................................................uucguugucgaugaaaccugU....................    3     1    Mm1
......................................................................uucguugucgCugaaaccugc....................    1     1    Mm1
......................................................................uucguugucgaugaaaccugc....................    261   0    Mm1
......................................................................uucguugucgGugaaaccugc....................    1     1    Mm1
......................................................................uucguugucgaugaaacAugc....................    1     1    Mm1
......................................................................uucguugucgaugCaaccugc....................    1     1    Mm1
......................................................................uucguugucgaugaGaccugc....................    1     1    Mm1
......................................................................uucguugucgaugaaaccuAc....................    1     1    Mm1
......................................................................uucguugucgaugaaacUugc....................    1     1    Mm1
......................................................................uucguugucgauUaaaccugcc...................    1     1    Mm1
......................................................................uucguugucgCugaaaccugcc...................    1     1    Mm1
......................................................................uucguugucUaugaaaccugcc...................    1     1    Mm1
......................................................................Nucguugucgaugaaaccugcc...................    4     1    Mm1
......................................................................uucgCugucgaugaaaccugcc...................    3     1    Mm1
......................................................................uucguCgucgaugaaaccugcc...................    1     1    Mm1
......................................................................uucguugucgaugaUaccugcc...................    1     1    Mm1
......................................................................uucguugucgaugaaaccuAcc...................    1     1    Mm1
......................................................................uuUguugucgaugaaaccugcc...................    1     1    Mm1
......................................................................uucguugucgGugaaaccugcc...................    4     1    Mm1
......................................................................uucguuAucgaugaaaccugcc...................    1     1    Mm1
......................................................................uucguugucgaugaaaccugcc...................    4934  0    Mm1
......................................................................uucguugucgUugaaaccugcc...................    7     1    Mm1
......................................................................uucguugucgaugaaaccCgcc...................    1     1    Mm1
......................................................................uuAguugucgaugaaaccugcc...................    2     1    Mm1
......................................................................uuGguugucgaugaaaccugcc...................    1     1    Mm1
......................................................................uCcguugucgaugaaaccugcc...................    1     1    Mm1
......................................................................uucguugucgaAgaaaccugcc...................    2     1    Mm1
......................................................................uucguugucgaugaaGccugcc...................    1     1    Mm1
......................................................................uucgAugucgaugaaaccugcc...................    1     1    Mm1
......................................................................uucAuugucgaugaaaccugcc...................    1     1    Mm1
......................................................................uucguugucgaugaaaccugcG...................    2     1    Mm1
......................................................................uucguugucgaugaaacGugcc...................    18    1    Mm1
......................................................................uAcguugucgaugaaaccugcc...................    2     1    Mm1
......................................................................uucguugucgaugaaaAcugcc...................    11    1    Mm1
......................................................................uucguNgucgaugaaaccugcc...................    1     1    Mm1
......................................................................uucguugucgaugaaacUugcc...................    28    1    Mm1
......................................................................uucguugucgaugaaCccugcc...................    1     1    Mm1
......................................................................uucguugucgaugaaaGcugcc...................    10    1    Mm1
......................................................................uucguugucgaugaaacAugcc...................    42    1    Mm1
......................................................................uucguugucgaugaaaccuCcc...................    1     1    Mm1
......................................................................uucguuUucgaugaaaccugcc...................    1     1    Mm1
......................................................................Gucguugucgaugaaaccugcc...................    1     1    Mm1
......................................................................uucguugucgaugaaaccugcU...................    35    1    Mm1
......................................................................uucguugucgaugaaaUcugcc...................    6     1    Mm1
```

Star Mature

```
ccucuauuagaaggaaucaggacauccaaauucaggucucacgacgauucgaacagucauuugaacuuuguucguugucgaugaaaccugccugcugguguccgagucgucga
......................................................................uucguugucAaugaaaccugcc.....................   1     1   Mm1
......................................................................uucguuNucgaugaaaccugcc.....................   1     1   Mm1
......................................................................uucguugucgauCaaaccugccu....................   1     1   Mm1
......................................................................uucguugucgaugaaaccugccA....................   57    1   Mm1
......................................................................uucguugucgGugaaaccugccu....................   10    1   Mm1
......................................................................uucguugucgaugaaaccugUcu....................   2     1   Mm1
......................................................................uucguugucgaugUaaccugccu....................   1     1   Mm1
......................................................................uucguugucgaugaaaGcugccu....................   8     1   Mm1
......................................................................uucgAugucgaugaaaccugccu....................   2     1   Mm1
......................................................................uucguugCcgaugaaaccugccu....................   2     1   Mm1
......................................................................uucguugucgaugGaaccugccu....................   2     1   Mm1
......................................................................Cucguugucgaugaaaccugccu....................   1     1   Mm1
......................................................................uucguugucgaugCaaccugccu....................   2     1   Mm1
......................................................................uucguugucgaugaaaccugcUu....................   5     1   Mm1
......................................................................uucguugucgaAgaaaccugccu....................   1     1   Mm1
......................................................................uucguugucgaugaaacAugccu....................   59    1   Mm1
......................................................................uucguugucgaugaaaccugccC....................   7     1   Mm1
......................................................................uAcguugucgaugaaaccugccu....................   3     1   Mm1
......................................................................uuUguugucgaugaaaccugccu....................   1     1   Mm1
......................................................................uucguugucgaugaaUccugccu....................   2     1   Mm1
......................................................................uucguugucgUugaaaccugccu....................   12    1   Mm1
......................................................................uucguugucgaugaaacGugccu....................   32    1   Mm1
......................................................................uucguugAcgaugaaaccugccu....................   1     1   Mm1
......................................................................uucguugucgaugaaaUcugccu....................   21    1   Mm1
......................................................................uucguugucgaugaCaccugccu....................   1     1   Mm1
......................................................................uucguugucgaugaaaccuUccu....................   2     1   Mm1
......................................................................uucguugucAaugaaaccugccu....................   2     1   Mm1
......................................................................uucguuguUgaugaaaccugccu....................   1     1   Mm1
......................................................................uucgCugucgaugaaaccugccu....................   3     1   Mm1
......................................................................uucguugucgaugaaaccuAccu....................   4     1   Mm1
......................................................................uucguugucgaCgaaaccugccu....................   1     1   Mm1
......................................................................Nucguugucgaugaaaccugccu....................   3     1   Mm1
......................................................................uucguugucUaugaaaccugccu....................   1     1   Mm1
......................................................................uucguNgucgaugaaaccugccu....................   2     1   Mm1
......................................................................uucguugucgaugaaacUugccu....................   42    1   Mm1
......................................................................uuGguugucgaugaaaccugccu....................   2     1   Mm1
......................................................................uucAuugucgaugaaaccugccu....................   1     1   Mm1
......................................................................uucguuguGgaugaaaccugccu....................   1     1   Mm1
......................................................................uuAguugucgaugaaaccugccu....................   1     1   Mm1
......................................................................uucguugucgCugaaaccugccu....................   12    1   Mm1
......................................................................uucguugucgaugaaaAcugccu....................   30    1   Mm1
......................................................................uucguugucgaugaaCccugccu....................   2     1   Mm1
......................................................................uucguugucgaugaaaccugccu....................   7406  0   Mm1
......................................................................uucguugucgaugaaaccAgccu....................   2     1   Mm1
......................................................................uucguugucgaugaaaccugccG....................   3     1   Mm1
......................................................................uucguugucgaugaaaccugcAu....................   1     1   Mm1
......................................................................uucguugucgauUaaaccugccu....................   1     1   Mm1
......................................................................uucguugucgaGgaaaccugccu....................   1     1   Mm1
......................................................................uucguuUucgaugaaaccugccu....................   1     1   Mm1
......................................................................uucguuguAgaugaaaccugccu....................   1     1   Mm1
......................................................................uucguugucgaugaaaccugccuU...................   21    1   Mm1
.......................................................................ucguugucgaugaaaccugcc.....................   14    0   Mm1
.......................................................................ucguugucgaugCaaccugcc.....................   1     1   Mm1
.......................................................................ucguugucgaugaaaccugccu....................   13    0   Mm1

...............................ucaggucucacgacgauucgaac...........................................................   1     0   Mm2
................................caggucucacgacgauucga.............................................................   9     0   Mm2
................................caggucucacgacgauucgaa............................................................   2     0   Mm2
................................caggucucacgacgauucgaac...........................................................   8     0   Mm2
................................caggucucacgacgauucgaaca..........................................................   4     0   Mm2
................................caggucucacgacgauucgaacag.........................................................   1     0   Mm2
................................caggucucacgacgauuAgaacagu........................................................   1     1   Mm2
..........................................................auuugaacuuuguucguugucgaugaaaccu........................   1     0   Mm2
....................................................................uCuucguugucgaugaaaccugccu....................   1     1   Mm2
.....................................................................guucguugucgaugaaaccug.......................   1     0   Mm2
.....................................................................guucguugucgaugaaaccugcc.....................   3     0   Mm2
.....................................................................Cuucguugucgaugaaaccugcc.....................   1     1   Mm2
.....................................................................guucguugucgaugaaaUcugccu....................   1     1   Mm2
.....................................................................guucguugucgaugaaaccugccu....................   10    0   Mm2
.....................................................................guucguugucgaugaaaccugccA....................   1     1   Mm2
```

```
                                    Star                                Mature
ccucuauuagaaggaaucaggacauccaaauucaggucucacgacgauucgaacagucauuugaacuuuguucguugucgaugaaaccugccugcugguguccgagucgucga
.....................................................................guucguugucgaugaaaccugcUu....................     2       1     Mm2
.....................................................................Cuucguugucgaugaaaccugccu....................     6       1     Mm2
......................................................................uucguugucgaugaaacU.........................     1       1     Mm2
......................................................................uucguugucgaugaaacc.........................     2       0     Mm2
......................................................................uucguugucgaugaaaccG........................     1       1     Mm2
......................................................................uucguugucgaugaaaccu........................     15      0     Mm2
......................................................................uucguugucgaugaaaccug.......................     68      0     Mm2
......................................................................uucguugucgaugaaacUug.......................     2       1     Mm2
......................................................................uucguugucgaugaaacUugc......................     2       1     Mm2
......................................................................uucguugucgaugaaaUcugc......................     2       1     Mm2
......................................................................uucguugucgaugaUaccugc......................     1       1     Mm2
......................................................................uucguugucgUugaaaccugc......................     1       1     Mm2
......................................................................uucguugucgaugaaaccugU......................     49      1     Mm2
......................................................................uucguugucgCugaaaccugc......................     1       1     Mm2
......................................................................uucguugucgaugaaacAugc......................     2       1     Mm2
......................................................................uucguugucgaugaaacGugc......................     2       1     Mm2
......................................................................uucguugucgaugaaaGcugc......................     1       1     Mm2
......................................................................uucguugucgaugaaaccugc......................     665     0     Mm2
......................................................................uucguugucAaugaaaccugc......................     1       1     Mm2
......................................................................uucguugucgaugaaaAcugc......................     3       1     Mm2
......................................................................uucguugucgGugaaaccugc......................     1       1     Mm2
......................................................................uucguugucUaugaaaccugc......................     1       1     Mm2
......................................................................uucguugucgaugaaaccugA......................     2       1     Mm2
......................................................................Nucguugucgaugaaaccugcc.....................     14      1     Mm2
......................................................................uucguugucgaugaaacAugcc.....................     162     1     Mm2
......................................................................uucguAgucgaugaaaccugcc.....................     1       1     Mm2
......................................................................uucguugucgaugaaacUugcc.....................     98      1     Mm2
......................................................................uucguugucUaugaaaccugcc.....................     4       1     Mm2
......................................................................uucguugucgaugaaaccuCcc.....................     1       1     Mm2
......................................................................uucguugucgaugaaaccugcc.....................     18058   0     Mm2
......................................................................uucguugucgaugaaaccugAc.....................     3       1     Mm2
......................................................................uucguugucgaugaaaccuUcc.....................     10      1     Mm2
......................................................................uucguugucgaugaaaccAgcc.....................     2       1     Mm2
......................................................................uucguugucgaugaaacGugcc.....................     71      1     Mm2
......................................................................uucguugucgGugaaaccugcc.....................     14      1     Mm2
......................................................................uucguugucgaugaaaccugUc.....................     4       1     Mm2
......................................................................uucguugucAaugaaaccugcc.....................     2       1     Mm2
......................................................................uucguugucgaugaaaccugcU.....................     193     1     Mm2
......................................................................uucguuguGgaugaaaccugcc.....................     1       1     Mm2
......................................................................uAcguugucgaugaaaccugcc.....................     5       1     Mm2
......................................................................uucguugucgaCgaaaccugcc.....................     2       1     Mm2
......................................................................uucguugucgaugaGaccugcc.....................     2       1     Mm2
......................................................................uucguuAucgaugaaaccugcc.....................     1       1     Mm2
......................................................................uucguugucgaugaaaUcugcc.....................     46      1     Mm2
......................................................................uuAguugucgaugaaaccugcc.....................     2       1     Mm2
......................................................................uucguugucgaugaaGccugcc.....................     1       1     Mm2
......................................................................uucguuguAgaugaaaccugcc.....................     1       1     Mm2
......................................................................uucguugucgaugaaaccugcA.....................     22      1     Mm2
......................................................................uucguugucgaugaaaGcugcc.....................     21      1     Mm2
......................................................................Cucguugucgaugaaaccugcc.....................     5       1     Mm2
......................................................................uucguuNucgaugaaaccugcc.....................     2       1     Mm2
......................................................................uucguugucgaAgaaaccugcc.....................     2       1     Mm2
......................................................................uucguugucgaugaaaAcugcc.....................     72      1     Mm2
......................................................................Aucguugucgaugaaaccugcc.....................     2       1     Mm2
......................................................................uucguugucgaugaaCccugcc.....................     1       1     Mm2
......................................................................uucguugucgaugaaaccCgcc.....................     1       1     Mm2
......................................................................uCcguugucgaugaaaccugcc.....................     4       1     Mm2
......................................................................uucguugucgaugaUaccugcc.....................     1       1     Mm2
......................................................................uuUguugucgaugaaaccugcc.....................     10      1     Mm2
......................................................................uucAuugucgaugaaaccugcc.....................     3       1     Mm2
......................................................................uucguugucCaugaaaccugcc.....................     4       1     Mm2
......................................................................uucguCgucgaugaaaccugcc.....................     4       1     Mm2
......................................................................uucguugucgauCaaaccugcc.....................     2       1     Mm2
......................................................................uuGguugucgaugaaaccugcc.....................     4       1     Mm2
......................................................................uucgAugucgaugaaaccugcc.....................     2       1     Mm2
......................................................................uucguugucgaugCaaccugcc.....................     2       1     Mm2
......................................................................uucguugucgaugGaaccugcc.....................     4       1     Mm2
......................................................................uucguugucgUugaaaccugcc.....................     21      1     Mm2
......................................................................uucguugucgCugaaaccugcc.....................     24      1     Mm2
......................................................................uucguuguUgaugaaaccugcc.....................     5       1     Mm2
```

Star Mature

```
ccucuauuagaaggaaucaggacauccaaauucaggucucacgacgauucgaacagucauuugaacuuuguucguugucgaugaaaccugccugcuggugucc gagucgucga
......................................................................uucguugucgaugaaaccuAcc.....................   6      1   Mm2
......................................................................uucguugucgaugaaaccugcG.....................   1      1   Mm2
......................................................................uucguugAcgaugaaaccugcc.....................   4      1   Mm2
......................................................................uucgGugucgaugaaaccugcc.....................   1      1   Mm2
......................................................................uucguugucgaugUaaccugcc.....................   3      1   Mm2
......................................................................uucUuugucgaugaaaccugcc.....................   1      1   Mm2
......................................................................uucguugucgauAaaaccugcc.....................   4      1   Mm2
......................................................................uucguugucgauUaaaccugcc.....................   1      1   Mm2
......................................................................uucguugucgaugaaaccugUcu....................   5      1   Mm2
......................................................................uucguugCcgaugaaaccugccu....................   1      1   Mm2
......................................................................uucguugucgaugaaaccAgccu....................   3      1   Mm2
......................................................................uucguugucgCugaaaccugccu....................   17     1   Mm2
......................................................................uucguugucgaugaGaccugccu....................   2      1   Mm2
......................................................................uucguugucgaugaaaccugAcu....................   1      1   Mm2
......................................................................uucguugucUaugaaaccugccu....................   2      1   Mm2
......................................................................uucguugucgaugaaaccugcAu....................   9      1   Mm2
......................................................................uucguugucgauCaaaccugccu....................   1      1   Mm2
......................................................................uucguugucCaugaaaccugccu....................   2      1   Mm2
......................................................................uucguugucgaugaaaccuCccu....................   2      1   Mm2
......................................................................uucguNgucgaugaaaccugccu....................   5      1   Mm2
......................................................................Aucguugucgaugaaaccugccu....................   4      1   Mm2
......................................................................uucCuugucgaugaaaccugccu....................   6      1   Mm2
......................................................................uGcguugucgaugaaaccugccu....................   1      1   Mm2
......................................................................uucguugucgaugaaaccuAccu....................   2      1   Mm2
......................................................................uucAuugucgaugaaaccugccu....................   3      1   Mm2
......................................................................uAcguugucgaugaaaccugccu....................   9      1   Mm2
......................................................................uucguugucgaugaaaccugccC....................   112    1   Mm2
......................................................................Cucguugucgaugaaaccugccu....................   4      1   Mm2
......................................................................uucguugucgaugGaaccugccu....................   3      1   Mm2
......................................................................uucguugAcgaugaaaccugccu....................   1      1   Mm2
......................................................................Nucguugucgaugaaaccugccu....................   8      1   Mm2
......................................................................uucguugucgaugaaacAugccu....................   185    1   Mm2
......................................................................uucgAugucgaugaaaccugccu....................   5      1   Mm2
......................................................................uucguugucgaugaaaccugccu....................   20379  0   Mm2
......................................................................uucguugucAaugaaaccugccu....................   5      1   Mm2
......................................................................uucguugucgaugaaaAcugccu....................   95     1   Mm2
......................................................................uucguugucgUugaaaccugccu....................   32     1   Mm2
......................................................................uucUuugucgaugaaaccugccu....................   2      1   Mm2
......................................................................uucguugucgaugaaacUugccu....................   107    1   Mm2
......................................................................uucguugucgaugUaaccugccu....................   5      1   Mm2
......................................................................uucguugucgaugaaaUcugccu....................   47     1   Mm2
......................................................................uucguugucgaugaaacGugccu....................   104    1   Mm2
......................................................................uucgGugucgaugaaaccugccu....................   1      1   Mm2
......................................................................uucguugucgaGgaaaccugccu....................   1      1   Mm2
......................................................................uucguuAucgaugaaaccugccu....................   2      1   Mm2
......................................................................uucguugucgaugaaGccugccu....................   3      1   Mm2
......................................................................uCcguugucgaugaaaccugccu....................   2      1   Mm2
......................................................................uucguugucgaAgaaaccugccu....................   4      1   Mm2
......................................................................uucguugucgaugaaaccuUccu....................   5      1   Mm2
......................................................................uucguugucgaugaaaccCgccu....................   2      1   Mm2
......................................................................uucguugucgaCgaaaccugccu....................   7      1   Mm2
......................................................................uucguugucgaugaaaccugccA....................   338    1   Mm2
......................................................................uuGguugucgaugaaaccugccu....................   3      1   Mm2
......................................................................uucguugucgGugaaaccugccu....................   25     1   Mm2
......................................................................uucguuUucgaugaaaccugccu....................   2      1   Mm2
......................................................................uuUguugucgaugaaaccugccu....................   7      1   Mm2
......................................................................uucguugucgaugaaaccugcUu....................   22     1   Mm2
......................................................................uucguCgucgaugaaaccugccu....................   6      1   Mm2
......................................................................uucguugucgaugaaaccugccG....................   90     1   Mm2
......................................................................uucgCugucgaugaaaccugccu....................   4      1   Mm2
......................................................................uucguugucgaugaaaGcugccu....................   24     1   Mm2
......................................................................uuAguugucgaugaaaccugccu....................   6      1   Mm2
......................................................................uucguugucgauAaaaccugccu....................   1      1   Mm2
......................................................................uucguugucgaugaaUccugccu....................   5      1   Mm2
......................................................................uucguuguUgaugaaaccugccu....................   13     1   Mm2
......................................................................uucguuNucgaugaaaccugccu....................   1      1   Mm2
......................................................................uucguugucgaugCaaccugccu....................   6      1   Mm2
......................................................................uucguugucgaugaaaccugccug...................   1      0   Mm2
......................................................................uucguugucgaugaaaccugccuA...................   5      1   Mm2
......................................................................uucguugucgaugaaaccugccCg...................   1      1   Mm2
```

```
                                Star                                  Mature
ccucuauuagaaggaaucaggacauccaaauucaggucucacgacgauucgaacagucauuugaacuuuguucguugucgaugaaaccugccugcugguguccgagucgucga
......................................................................uucguugucgaugaaaccugccuU...................    82    1    Mm2
......................................................................uucguugucgaugaaaccugccuUc..................    2     1    Mm2
.......................................................................ucguugucgaugaaaccugc......................    1     0    Mm2
.......................................................................ucguugucgaugaaaccugcc.....................    49    0    Mm2
.......................................................................ucguugucgaugaaaccugccu....................    60    0    Mm2
.......................................................................ucguugucgaCgaaaccugccu....................    1     1    Mm2
.......................................................................uUguugucgaugaaaccugccu....................    1     1    Mm2
.......................................................................ucguugucgauUaaaccugccu....................    1     1    Mm2
.......................................................................ucguugucgaugaaaccuUccu....................    1     1    Mm2
.......................................................................ucguugucgaugaaaccugcUu....................    1     1    Mm2
.......................................................................ucguugucgaugaaaccugccA....................    1     1    Mm2
........................................................................cguugucgaugaaaccugccu....................    8     0    Mm2
........................................................................cguugucgaugaaaccugccA....................    1     1    Mm2
.........................................................................guugucgaugaaaccugcc.....................    1     0    Mm2
.........................................................................guuAucgaugaaaccugccu....................    1     1    Mm2
.........................................................................guugucgaugaaaccugccu....................    3     0    Mm2
```
